# Celebrating the 15th anniversary of the Royal Society Newton International Fellowship

**DOI:** 10.1098/rsta.2023.0326

**Published:** 2024-10-23

**Authors:** Leonette Annan

**Keywords:** Newton International Fellowship, grants programme, Royal Society

In 2008, the Royal Society established the *Newton International Fellowships* programme, which has now supported over 650 early career researchers from over 50 countries, with over £72.5 million of funding allocated by the Royal Society since 2008.

The overarching aim of the *Newton International Fellowship* programme is to attract emerging talent to the UK, to help develop the careers of the fellows and to build a globally connected, mobile research and innovation workforce. The specific objectives have been to:

— attract talented international early career researchers to establish and conduct their research in the UK;
— support early career researchers to pursue high-quality and innovative lines of research;
— provide opportunities to acquire new skills and knowledge through training and career development;
— foster long-term relationships through networking opportunities and the *Newton International Fellowships* alumni programme.

The scheme was initially proposed and developed in 2007 to act on the recommendation of the UK’s Global Science and Innovation Forum (responding to announcements by then and previous Prime Ministers and Alistair Darling in his former role), namely ‘to establish a new fellowship scheme to attract the very best researchers to the UK from overseas and to manage the alumni of that scheme’. On 4 June 2008, the *Newton International Fellowship* programme was officially launched at an event involving the then Minister of State for Science and Innovation, Ian Pearson.

The *Newton International Fellowships* are a cross-academy scheme. They were originally established in collaboration with the British Academy and the Royal Academy of Engineering. In 2010, the Royal Academy of Engineering left the scheme, while in 2015, the Academy of Medical Sciences joined. This scheme therefore covers a broad range of subject disciplines.

The *Newton International Fellowships* continue to be a key component of the Royal Society’s strategic focus on international collaboration. Throughout its long history, the society has promoted science as a global endeavour and has always been a voice for science globally. It has worked to bring excellent international science to the UK, and to promote UK science internationally; and by working bi- and multi-laterally to remove barriers to international scientific cooperation and promote academic freedom.

Feedback we have had from past *Newton International* fellows in the last review of the scheme has highlighted the positive impact this opportunity has had on their careers. Respondents from a survey conducted as part of their review revealed past fellowship holders considered that the *Newton International Fellowship* led to faster career progression and an enhancement in how they were perceived by the research community.

The papers in this theme issue aim to illustrate and celebrate the research undertaken during, and facilitated by, the Royal Society’s *Newton International Fellowship* programme. As a commemorative issue, this theme issue features authors from the first fellowship cohorts and shows how the work undertaken during their fellowships has influenced their research today. The fellowships will continue to be an essential component of the Royal Society’s grants and international programmes.

We are grateful to all who have contributed to this theme issue and to all those who during the last 15 years have helped and promoted the success of the *Newton International Fellowships* programme.

## Data Availability

This article has no additional data.

